# Pediatric chronic myeloid leukemia in myeloid blast crisis

**DOI:** 10.4322/acr.2023.426

**Published:** 2023-04-13

**Authors:** Biswajit Dey, Anirban Dutta

**Affiliations:** 1 North Eastern Indira Gandhi Regional Institute of Health and Medical Sciences, Department of Pathology, Shillong, Meghalaya, India; 2 University of Hawaii John A. Burns School of Medicine, Department of Pediatrics, Honolulu, Hawaii, United States of America

**Keywords:** Blast Crisis, Leukemia, Myelogenous, Chronic, BCR-ABL Positive, Leukemia, Myeloid, Leukemia, Myeloid, Acute, Pediatrics

## Abstract

Chronic myeloid leukemia (CML) accounts for 2-3% of childhood leukemias. About 5% of cases present in a blastic phase of CML which clinically and morphologically mimics more common acute leukemias of childhood. We report a case of a 3-year-old male who presented with gradual onset swelling of the abdomen and extremities along with generalized weakness. Examination revealed massive splenomegaly, pallor, and pedal edema. Initial workup showed anemia, thrombocytopenia, and leukocytosis (120,000/uL) with a blast percentage of 35%. Blasts were positive for CD13, CD33, CD117, CD34 and HLA-DR, and stained negative for Myeloperoxidase and Periodic Acid Schiff. Fluorescence in situ hybridization was positive for b3a2/e14a2 junction BCR-ABL1 transcript and negative for RUNX1-RUNX1T1/t(8;21), clinching the diagnosis of CML in myeloid blast crisis. The patient expired within 17 days of diagnosis and initiation of therapy.

## INTRODUCTION

Chronic myeloid leukemia (CML) is a clonal, myeloproliferative malignancy accounting up to 15% of all adult leukemias.^1^ However, it is a rarer disease in children, attributing around 2-3% of childhood leukemias.^1,2^

CML is classically staged into three progressive phases - chronic, accelerated, and blastic. The blastic phase is defined as the presence of at least 20% blasts (>30% according to European LeukemiaNet [ELN] recommendations) in the blood or bone marrow or the presence of blasts in extramedullary sites.^2^ In children, the incidence of CML presenting in a blastic phase is reported to be less than 5%, compared to 10% in adults.^3^

CML in the blastic phase (CML-BP) behaves clinically like acute leukemia and resembles morphologically acute leukemia in children.^4^ Hence such cases require a thorough evaluation. Here we report a rare case of a 3-year-old male presenting in the blastic phase, posing clinical and diagnostic challenges.

## CASE REPORT

A 3-year-old male child presented with a history of gradual swelling of the abdomen for the past eight months and swelling of the hands and feet with a generalized weakness for the past two months. There was no history of fever, rash, bleeding, or jaundice. The patient’s appetite, bowel and bladder habits were normal. There was no history of similar episodes or recurrent infections in the past. According to his growth charts, the child’s growth rate had been within the normal percentile range. Physical examination revealed the presence of pallor, pedal edema, and massive splenomegaly (6 cm from the left costal margin) but no significant lymphadenopathy or palpable hepatomegaly. The remainder of the physical examination was within normal limits.

**Figure 1:**
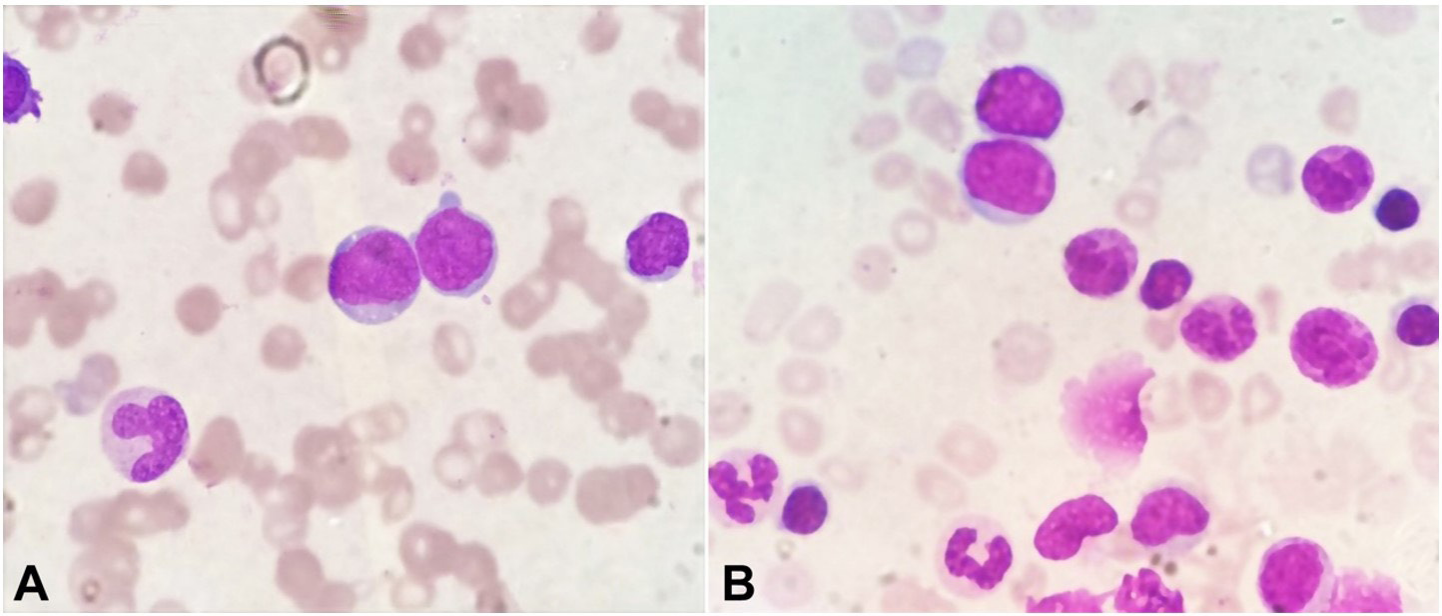
Photomicrograph of **A -** peripheral blood smear; and **B -** bone marrow aspirate showing blast with coarse nuclear chromatin and prominent nucleoli. Some of the blasts had indented nuclei. (Leishman stain, x1000).

Initial laboratory investigations showed anemia (hemoglobin 6.6 gm%, reference range [RR]: 10.5-13.5 gm%), thrombocytopenia (platelet count 30,000/µL, RR: 2,00,00-4,50,000/µL), and leukocytosis (WBC count 120,000/µL, RR: 4,900-12,800/µL). A shift to the left was noted on differential leukocyte count with a blast count of 35% and basophilia (4%). The blast cells were round to oval, having coarse nuclear chromatin and prominent nucleoli, with a few cells containing indented nuclei (Figure 1A). Blast cells had a high nuclear-cytoplasmic (N:C) ratio, but no Auer rods were observed.

Bone marrow aspirate showed suppressed erythropoiesis, subtle megaloblastic reaction, and mild dyspoiesis. Blasts accounted for 24% of the cellularity, with a myeloid: erythroid ratio of 14:1 (Figure 1B). Megakaryocytes were smaller and hyposegmented with dwarf morphology. Cytochemistry demonstrated blasts staining negative for both Myeloperoxidase (MPO) and Periodic Acid Schiff (PAS).

**Figure 2:**
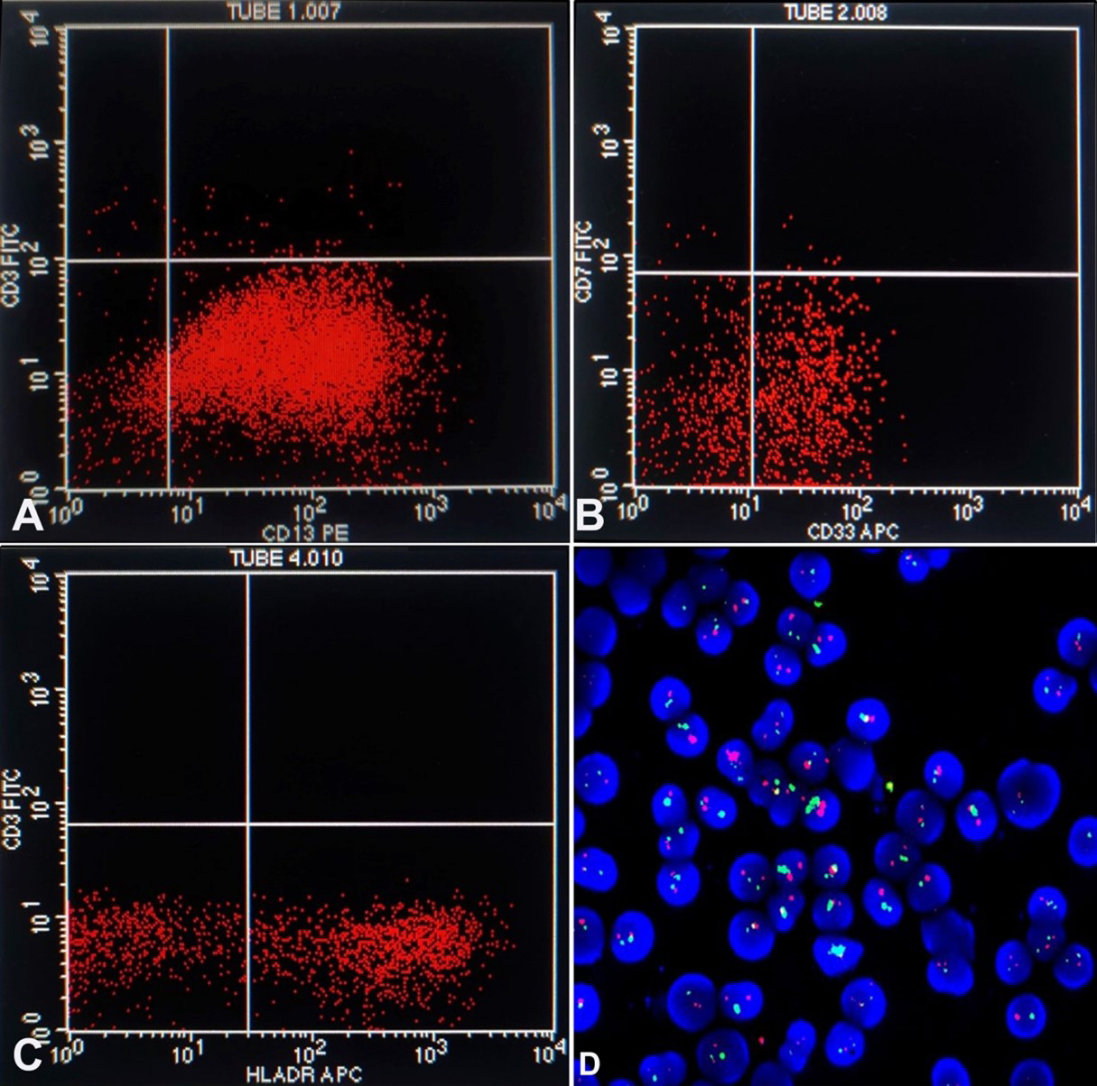
Flow cytometry showing blasts positive for: **A -** CD13; **B -** CD33; **C -** HLA-DR; **D -** FISH with dual fusion probe showing positive for *BCR/ABL1* rearrangement.

Flow cytometry of the bone marrow aspirate was performed using BD FACScalibur, and blasts were gated by CD45/Side Scatter gating strategy for immunophenotyping (IPT). On IPT, the blasts were positive for CD13, CD33, CD117, CD34 and HLA-DR but were negative for Tdt, CD3, CD4, CD7, CD19, and CD20 (Figure 2A, 2B and 2C). IPT findings confirmed the myeloid nature of the blasts.

Fluorescence in situ hybridization (FISH) with dual fusion probe on peripheral blood (PB) sample for BCR/ABL gene rearrangement was performed considering the probability of acute leukemia vis-a-vis CML-BP. Abnormal clones with BCR-ABL1 (Breakpoint cluster region - Abelson gene) fusion were noted in 30.5% of cells (Figure 2D). FISH was negative for RUNX1-RUNX1T1/t(8;21) fusion gene, further undermining a diagnosis of AML. He had the b3a2/e14a2 junction BCR-ABL1 transcript, which encodes p210. Karyotyping could not be done as the facility was not available in the institute.

Correlating the clinical presentation of massive splenomegaly, the patient’s hematological parameters, IPT, and the presence of p210 BCR-ABL1 transcripts, a final diagnosis of CML in myeloid blast crisis was made.

The patient was started on Imatinib mesylate 400 mg/m^2^ and planned to be re-evaluated at the end of three months, but he died due to febrile neutropenia after 17 days of initiation of therapy.

## DISCUSSION

CML-BP is the last stage of CML's progression, and it acts like acute leukemia, with rapid progression and may morphologically resemble acute leukemia in children.^4^ CML blast transformation is lymphoid in 30% of cases and myeloid in 70% of cases.^4^ There are only a few isolated case reports of pediatric CML either presenting or transforming into myeloid blast crisis. An extensive search in PubMed and Google scholar with the keywords ‘pediatric’, 'chronic myeloid leukemia’ and ‘myeloid blast crisis’ revealed 4 cases in English language, which had all the laboratory and clinical details.^1,4-6^ The details of these 4 cases are summarized in Table 1.

**Table 1:** Summary of pediatric chronic myeloid leukemia in myeloid blast crisis

Ref	# Of cases	Age years	Sex	Percentage of blasts	Confirmation of Myeloid nature of blasts	Outcome
1	1	4	F	20% in PBS	FCM	NM
4	1	4	M	38% in PBS	FCM	Doing well after 5 months of therapy
				36% in BM		
5	1	5	M	85.5% in cerebrospinal fluid	FCM	Doing well after 3 years of diagnosis
6	1	9.1	F	31%	FCM	Complete molecular response after 10 months of therapy

PBS = Peripheral blood smear; BM = bone marrow; FCM = flow cytometry; NM = not mentioned; ref = reference number.

In the present case, the myeloid nature of the blasts was confirmed by flow cytometry. The blasts were positive for myeloid markers CD13, CD33, and CD117. All the lymphoid markers, including B-lineage and T-lineage markers, were negative.

The biology of pediatric CML to BP progression is still unknown; however, it is thought to be comparable to that of adults. Because of its inherent tyrosine kinase activity, the Philadelphia (Ph1) chromosome and the resultant BCR-ABL1 fusion gene induce granulocytic as well as blast proliferation in leukemic stem cells.^1,2^ This is partly because BCR-ABL1 suppresses c-Jun, a monopoiesis-promoting transcription factor in both CML neutrophils and blasts.^1^ As a result, in CML, the BCR-ABL1 fusion gene is expected to be detected in mature neutrophils also unlike the blasts of de novo AML.^1^ Furthermore, mature eosinophils and basophils also possess the BCR-ABL1 fusion gene, which causes them to proliferate in CML.^1^ BCR-ABL1 in CML usually encodes p210 fusion transcript (b3a2/e14a2 junction).^4,7^ It is also seen in 2-4% of pediatric acute lymphoblastic leukemia cases and is usually p190.^7^ In the present case, BCR-ABL1 gene rearrangement was demonstrated by FISH and p210 was demonstrated by RT-PCR. However, BCR-ABL1 was demonstrated in only 30.5% of the PB cells. This could be attributed to the sample tested and the technique used. Although the common practice is to use PB instead of bone marrow aspirate to detect BCR-ABL1 in CML, it has been questioned due to relevant differences at specific cut-offs that might influence the sensitivity of molecular testing in CML. FISH is a faster method for BCR-ABL1 detection, but it suffers from human observer bias.^8^

About 0.5-3% of all AML cases also have the BCR-ABL1 fusion gene.^9^ “AML with BCR-ABL1” has been included as a provisional entity in the updated World Health Organization (WHO) 2016 classification of myeloid malignancies.^10^ Since no precise criteria have been developed, it can be challenging to distinguish between “de novo AML with BCR-ABL1” and “myeloid CML-BP” in many circumstances.^9^ In the present case, splenomegaly and basophilia favored a diagnosis of “CML-BP ” over ‘AML with BCR-ABL1’. Moreover, deletions of *IKZF1* and *CDKN2A* have been reported in AML with BCR-ABL1 and seem to be absent in the myeloid blast crisis of CML.^9^ These could help establish the diagnosis in complex cases.

It is of paramount importance to differentiate CML-BP from AML with BCR-ABL1 as these have different treatment modalities.^9^ CML-BP is treated by tyrosine kinase inhibitor followed by an early allogeneic stem cell transplant, whereas AML with BCR-ABL1 is treated with intense induction chemotherapy.^9^ The outcome with current tyrosine kinase inhibitors is often poor, and survival after presentation with CML-BP is generally less than 1 year. The present patient died after 17 days of initiating therapy due to febrile neutropenia.

## CONCLUSION

CML-BP is a rare presentation in the pediatric demographic mimicking more common diseases in that age group, including AML. Physicians should keep a high index of suspicion as treatment, as well as prognosis for CML, is distinct from these other diseases.
